# Complementary medicine among individuals experiencing homelessness in Switzerland: a quantitative and qualitative descriptive study

**DOI:** 10.1186/s12906-024-04727-4

**Published:** 2025-05-06

**Authors:** Véronique S. Grazioli, Evelyne Hangartner, Patrick Bodenmann, Luana Schaad, Léa Grosjean, Justin Nikles, David J. T. Campbell, Laurent Hyvert, Tshahé Anongba Varela, Susan E. Collins, Caroline Leblanc, Christine Loignon, Chantal Berna

**Affiliations:** 1 Department of Vulnerabilities and Social Medicine, Center for Primary Care and Public Health, Chair of Medicine for Vulnerable Populations, University of Lausanne, Lausanne, Switzerland; 2 Departments of Medicine, Community Health Sciences & Cardiac Sciences, Cumming School of Medicine, University of Calgary, Calgary, Canada; 3 Sleep-In Association, Renens, Switzerland; 4 Department of Psychology, Washington State University, Pullman, USA; 5 Family Medicine Department and Emergency, University of Sherbrooke, Sherbrooke, Canada; 6 Center for Integrative and Complementary medicine, Department of anesthesiology, Lausanne university hospital, Lausanne, Switzerland

**Keywords:** People experiencing homelessness, Complementary medicine, Integrative medicine, Switzerland

## Abstract

**Background:**

People experiencing homelessness (PEH) are disproportionately affected by health issues yet remain underserved by the health care system. Emerging findings suggest that complementary medicine (CM) approaches might help address the low access to earlier treatment and the complex needs of this population. Very little research has explored this topic in Europe. Thus, this study aimed to explore perceptions, experiences, and interests in CM among PEH in Switzerland.

**Methods:**

Participants (*N* = 123) were PEH in French-speaking Switzerland who completed a questionnaire assessing their use of and interest in CM. A subsample of the survey participants (*n* = 18) and 14 professionals working in the homeless-serving sector participated in semi-structured interviews exploring perceived utility of CM for PEH. Descriptive statistics and conventional content analysis were used to analyze quantitative and qualitative data, respectively.

**Results:**

Quantitative findings showed that despite high levels of interest in CM, less than 30% of participants reported using CM at least once in the previous 6 months. The five CM modalities with the highest interest were osteopathy (61.5% interested or very interested), therapeutic massage (59.2%), nutritional supplements (57.1%), music therapy (50.8%), and acupuncture (49.1%). The qualitative findings reinforced the substantial interest in CM. They revealed that CM approaches were perceived as useful to mitigate some health and social issues frequently encountered in this population, ultimately contributing to improved health and well-being. Participants made recommendations for practices that would help engage and retain PEH in a CM program, such as making it accessible and equitable, and following certain steps to earn the trust of PEH. Finally, a minority of participants questioned the relevance of a CM program for this population, arguing instead that more urgent social and conventional medical needs must be addressed first.

**Conclusions:**

Taken together, these findings suggest that integrative medicine, which incorporates conventional and complementary approaches to medical and social services may represent a suitable offering for PEH to address their competing bio-psycho-social needs. Using a community-based participatory approach to co-develop such a program might help to ensure effectiveness and thereby increase health equity.

**Supplementary Information:**

The online version contains supplementary material available at 10.1186/s12906-024-04727-4.

## Background

Recognized as a fundamental right in the Universal Declaration of Human Rights established by the United Nations, adequate housing contributes to individuals’ dignity, inclusion, well-being and security [1]. Despite this recognition, homelessness represents a growing public health issue worldwide due to structural factors, such as increasing socioeconomic inequality and the widespread affordable housing crisis [1]. It is estimated that 150 million people are homeless worldwide [2]. While definitions of homelessness vary, commonly accepted definitions include people who are: sleeping outdoors or in tents (i.e., sleeping/living rough), sleeping in emergency accommodation or transitional housing, staying in institutions (such as hospitals and prisons) with no place to live outside of the institution, living in non-conventional dwellings or in conventional housing with family or friends due to lack of housing (i.e., couch surfing) [3]. A recent report established by the European Social Policy Network indicates that the prevalence of homelessness increased drastically over the past decades in most European countries [3]. Despite the lack of official statistics, local monitoring and reports indicate that homelessness also represents a public health issue in Switzerland. A recent study revealed that up to 730 individuals were estimated to be homeless on a given night in 2021 in Geneva city (for 204,784 residents in 2021) [4]. In the same year, it was estimated that about 250 individuals were homeless in Lausanne (146,910 residents in 2021) [5], although internal monitoring reports from local emergency shelters (i.e., describing the number of different PEH using their facility and the number of refusals per year) suggested that this number may be underestimated [6].

Epidemiological research consistently highlights that people experiencing homelessness (PEH) face inequities across a wide range of health issues [7–9]. Besides being disproportionately affected by infectious illnesses (e.g., tuberculosis, HIV) [10], chronic medical conditions (e.g., heart disease, stroke) [11–13] and mental-health challenges (e.g., depression, anxiety) [14, 15], they are at higher risk of dealing with substance use disorders [16, 17] and being exposed to aggressions, injuries and intoxications [18–20]. As a result, premature mortality is common in this population, with all-cause mortality being up to 3.5 times higher compared to the general population [21, 22] .

Despite their important health care needs, this population generally remains underserved by health care systems [8, 21]. Due to a lack of access to preventative and outpatient services, such as primary care physicians, PEH often consult medical providers late in their illness and quit treatment programs prematurely, resulting in disproportionate use of emergency and inpatient services and ultimately unmet medical needs [8, 23]. A previous study involving patients from health centers in the USA documented that PEH had twice the odds of having unmet medical care needs and an emergency department (ED) visit in the past year, compared to those who were housed [8]. Past research described various barriers to health care in this population, such as practical issues (e.g., lack of insurance), competing basic needs (e.g., ensuring food and shelter) and emotional barriers (e.g., fear of stigmatization, negative experiences with mainstream health care services) [23–25].

In line with the commonly stated goal of improving health equity, health care systems must take actions to facilitate access to care for those experiencing homelessness. Since access to conventional health care services remains suboptimal for this population, the same can be assumed of access to other modalities of care, such as complementary and integrative medicine. The National Center for Complementary and Integrative Health [26], a branch of the US National Institutes of Health (NIH) defines Integrative Medicine (IM) as “bringing conventional and complementary approaches together in a coordinated way treating the whole person.” According to the NIH, complementary medicine (CM) refers to “approaches that are not typically part of conventional medical care or that may have origins outside of usual Western practice used together with conventional medicine [26].” CM are typically categorized into five categories: biologically based therapies (e.g., food supplements, phytotherapy etc.); complete medical systems (e.g., homeopathy); mind-body-based methods (e.g., meditation, music therapy); manipulative and body-based methods (e.g., therapeutic massage); and energy therapies (e.g., Qi gong). The incorporation of CM in primary care settings may be particularly pertinent for PEH, given that they address patients’ health care at the somatic, psychological, spiritual, and social levels, which may be a promising means to help manage the complex and competing needs of this population [27–29]. Notably, in certain conditions, such as chronic pain, CM is recommended as first-line treatments, yet it appears that there are some discrepancies in access depending on incomes [30, 31].

In fact, emerging findings from North America and Ireland indicate that CM, such as relaxation practices, mindfulness-based meditation, acupuncture, or music therapy are feasible, accepted and associated with improvements in well-being and in health in populations of PEH [28, 29, 32–35]. For instance, a recent pilot study evaluated a 4-week program teaching CM approaches (e.g., meditative practices, art therapy, tai chi) among 42 military veterans (most of them recently homeless) within a mental health residential rehabilitation program in the USA. Findings revealed that the program was well received and led to significant improvements in physical and mental domains of quality of life [33]. Other pilot studies tested the feasibility of implementing mindfulness-based meditation among men experiencing homelessness in Ireland [36], women and young children experiencing homelessness [37] and youth experiencing homelessness in shelters in the USA [38]. In addition to confirming the feasibility of implementing such an approach, qualitative findings revealed that participants perceived improvements in their own health [37], while pre-post quantitative findings showed significant improvements in mental health scores (e.g., anxiety, depression, and stress) [36, 38]. Finally, a recent narrative review examining music therapy among PEH revealed that this approach was often well received and followed by improvements in mental health, including stress reduction, improved self-esteem and self-efficacy [35].

Despite these promising early findings, the research exploring experiences, interest, and access to CM among PEH in Europe is very scarce, and non-existent in Switzerland. Such research is a crucial first step, given the increasingly recognized principle that health care interventions must arise from local contexts and be developed based on the views and experiences of those concerned, to ensure an equitable, inclusive, and effective intervention [39]. In response, this study, conducted among PEH and professionals in the homeless serving sector, aimed to provide a comprehensive assessment of the experiences, perceptions, and interests in CM of PEH in the Canton of Vaud, in Switzerland using quantitative and qualitative approaches.

## Methods

### Study design and setting

The study was conducted in the French-speaking Canton of Vaud in Switzerland (826,380 residents, approx. 9.4% of the Swiss population). In Switzerland, the healthcare system is largely funded by compulsory private insurance. Basic insurance premiums are covered by the Canton of Vaud when individuals’ incomes are minimal. However, while health insurance is compulsory, some people, including undocumented immigrants and those who have been excluded from society may not have the ability to purchase or obtain health insurance, making access to anything but emergency care completely inaccessible. To address this gap, there are a few charitable health-related programs to provide access to services people may need. Finally, basic health insurance does not cover most CM approaches (covered by additional non-compulsory insurances).

We conducted quantitative and qualitative assessments to meet the study aims. The quantitative strand was comprised of a paper-pencil cross-sectional survey administered by research staff to PEH. The survey quantified participants’ prior use and interest in CM. For the qualitative strand, we undertook a qualitative descriptive study to summarize participants’ experiences and perceptions. Specifically, we conducted semi-structured interviews in a subsample of PEH as well as professionals in the homeless-serving sector (professionals). Findings were interpreted in the light of both methods. This study was part of a larger research project aimed at describing perceptions, experience, and needs regarding both conventional and complementary medicine. Findings on conventional medical needs are presented in a separate manuscript [40]. The study was conducted in accordance with the Declaration of Helsinki and all procedures were approved of by the ethics committee of the Canton of Vaud (project number: ID 2022—249). Moreover, staff are used to working with the target population and have demonstrated great flexibility and a capacity for continuous adaptation.

### Participants

Participants in the quantitative survey (*N* = 123) were PEH in the Canton of Vaud. Inclusion criteria were: (1) being ≥ 18 years old; (2) experiencing currently homelessness according to the categories of the ethos-light typology (i.e., living rough, in emergency accommodation, in accommodation for the homeless, in institutions, in non-conventional dwellings due to lack of housing or in conventional housing with family or friends due to lack of housing) [3]; (3) being fluent in a language spoken by the research staff (i.e., French, English, Portuguese, Arabic) or having an interpreter available at the time of the survey. Exclusion criteria included being unable to provide informed consent due to language barriers, intoxication, or cognitive challenges, and having already completed the questionnaire. It has been estimated that about 250 individuals are experiencing homelessness in Lausanne area [5]. Based on this available estimation, we aimed to recruit 125 PEH to be as representative as possible of the full population.

Some of those who completed the questionnaire were purposively sampled and invited to participate in in-depth semi-structured interviews. For purposive sampling of PEH, we used a quota sampling approach according to health insurance status (insured vs. not) and residency status (permit vs. no permit). We also ensured that gender proportions were representative of the ones known in the target population.

We also invited professionals to participate in semi-structured interviews. Incorporating their perspectives was deemed critical to reach a comprehensive understanding of the topic and explore how the implementation of CM interventions should be carried out to ensure feasibility. Inclusion criteria among professionals were: being ≥ 18 years old; providing informed consent; working/having worked with PEH. For professionals, we used a quota sampling according to professions (i.e., night watcher in emergency shelters; healthcare workers including CM practitioners, other professionals involved with the target population).

### Procedures

Recruitment took place from May to August 2022 across 10 homeless-serving institutions (six emergency night shelters, a community health center, and three day-shelters) in the four largest cities in the canton of Vaud, where most PEH reside (Lausanne, Yverdon-les-Bains, Vevey and Nyon). Flyers describing the study were posted in the institutions, then participants were approached by the research team, with support of the local employees. Trained research staff conducted information sessions with interested PEH. While the questionnaire was initially designed to be self-administered by the PEH, piloting revealed this was not realistic and required active questioning by a research team member. When participants communicated in a language not spoken by the research team (i.e., Romanian), a community interpreter was involved in the assessment. All participants provided written informed consent to participate, and the questionnaire completion took approximately 40 min.

Following the quota sampling strategy, research staff planned semi-structured interviews with eligible participants after the completion of the questionnaire. Among PEH, the interviews were conducted face to face within a partner institution. The semi-structured interview lasted between 30 and 60 min. Among professionals, recruitment was conducted with support from heads of the homeless-serving institutions who promoted study participation amongst their teams. Eligible professionals were contacted by the research staff to participate in semi-structured interviews. Similarly, invitations followed the quota sampling protocol. Interviews were conducted face-to-face within the institutions or by phone, depending on staff availability. Professionals provided informed written consent prior to participating in the interview. The interview lasted between 30 and 60 min.

The qualitative interviews were conducted by a member of the research team with experience in qualitative research (a female Caucasian PhD student, a bachelor-level male Caucasian medical student, a PhD female Caucasian senior scientist), under supervision of the first author (PhD female Caucasian senior scientist). Qualitative interviews were conducted until data saturation was reached, i.e., when no new ideas were expressed in the interviews. The PEH participants received a 10-CHF (∼ 11 USD) grocery gift card for completing the questionnaire and an additional 20-CHF (22 USD) one for taking part in the semi-structured interview.

### Measures

#### Quantitative measures

The content of the questionnaire was reviewed and edited by all authors, professionals from two local homeless-serving institutions and by a local association that promotes literacy (i.e., “Lire et Écrire”) to ensure its relevance, readability and understandability among the target population. The questionnaire was translated and back-translated from French to English, Portuguese, Arabic by research staff fluent in these languages. The same procedure was conducted with medical students fluent in Romanian for the questionnaire in this language (see Appendix 1 for the questionnaire).

##### Demographic variables

Single items were used to assess sociodemographic information (e.g., age, gender, nationality, housing status).

##### Recent use of CM

Participants were asked to indicate which CM modalities they had used in the past 6 months from a list of 17 CM approaches which covered the 5 above-mentioned categories; (i.e., biologically based, mind-body-based and body-based therapies; energy therapies; alternative medical systems). These items were adapted from a previous study conducted among PEH [28]. The description of each CM modality was systematically given by the member of the trained research staff who hetero-administrated the questionnaire. Participants who answered having used at least one CM were subsequently asked to indicate how they accessed it (i.e., paid cash, was provided free-of-charge, paid for by health insurance, other).

##### Interest in CM

To assess interest in CM, participants were asked to indicate which CM modalities they would be willing to use from the same list of 17 CM.

#### Qualitative measures

We conducted semi-structured interviews with PEH, using an interview guide to explore perceptions of CM, experiences with CM, interests in CM and recommendations about how to develop a novel CM offering for the target population. The interview guide was developed by the research team based on literature and was validated by the whole team and partners of the research project (i.e., health care workers and night shelter watchers involved with the target population). If needed, research staff conducting the interview asked participants’ perceptions of specific CM (listed in the quantitative questionnaire, at least one of each category) after explaining precisely what the CM entails. The same dimensions were explored among professionals (see Appendix 2 for the semi-structured interview guides).

### Data analysis

#### Quantitative analysis

The questionnaire data were recorded in REDCap© by a research assistant. A second researcher double-checked data entry to minimize errors. Descriptive statistics were used to summarize the data. Descriptive statistics were performed using IBM SPSS (version 27).

#### Qualitative analysis

The data were analyzed using inductive conventional content analysis with the constant comparative process [41, 42]. The interviews were audio-recorded and transcribed verbatim by a professional transcriptionist. To improve the rigor and credibility of our results, three researchers were heavily involved in the analysis, which included interview debriefing, transcript coding, and data display and interpretation. First, to develop the codebooks (one for professionals and one for PEH), two researchers (EH: PhD candidate and LS: MD candidate) with experience in qualitative research completed initial coding independently. The researchers pooled their codes to reduce redundant, idiosyncratic, or common codes to generate a comprehensive codebook. A third senior researcher (VG: PhD) tested the codebook, and provided feedback, resulting in codebook adaptations. Then, two researchers (VG and LS) double-coded 10% of the interviews until an inter-judge agreement of at least 80% was achieved. Then, a single researcher (VG) conducted focused coding using Atlas.ti software version 9.0.3. Once all transcripts were coded and analyzed, VG, LS and EH met to identify the overarching topic areas across the interviews and observations notes.

## Quantitative results

### Demographics

The sample for the quantitative survey included 123 (mean age = 41.26, *SD* = 12.43, 91.1% self-identified as men). On average, participants reported having been homeless for 36.5 months (*SD* = 73.88; median = 5, IQR = 22). Table 1 displays additional descriptive data.

**Table 1:** Descriptive Statistics for the Study Sample (*N* = 123)

Variables	% or mean (SD)
Nationality	
Swiss	6.5%
European^1^	52%
Non-European^2^	41.5%
Residency permit^3^	
Undocumented	68.5%
Permit B	9%
Permit C	2.7%
Permit G	1.8%
Permit L	1.8%
Permit N	4.5%
No answer	9%
Highest educational level	
Mandatory school not completed	14.6%
Mandatory school (11 years)	24.4%
High school	24.4%
Vocational school	19.5%
University^4^ College	15.4%
Missing	1.6%
Housing status over the past 2 weeks	
Living rough	20.3%
In emergency accommodation	60.2%
In accommodation for the homeless	2.4%
In institution	0.8%
In non-conventional dwelling due to lack of housing	5.7%
In conventional housing (relatives, lack of housing)	5.7%
Own housing^5^	3.3%
Health insurance	
Hold a health insurance	49.2%
No health insurance	49.2%
No answer	1.6%

Note.^1^Examples of European country of origin includes France or Spain. ^2^Examples of non-European country of origin include Nigeria or Algeria. ^3^B permit authorizes non-Swiss citizen to stay in Switzerland for a specific purpose (e.g., work); C permit allows its holders to stay in Switzerland permanently; G permit allows cross-border working (i.e., people employed in Switzerland but living in a European country); L permit allows workers from the European Union to settle in Switzerland for a period of less than one year, with or without an employment contract; N permit: allows to stay in Switzerland while awaiting a decision on the asylum application. ^4^College = bachelor level, whereas University = Master level. ^5^Four participants lost their own housing in the past 2 weeks

### Use of CM

As shown in Table 2, most participants reported that they hadn’t used any form of CM in the past 6 months. The most frequently used CM were therapeutic massage, meditation, nutritional supplements, and music therapy. Participants who had used at least one CM in the past 6 months (*n* = 36) reported that they paid for it using cash (*n* = 17), receiving the treatment for free (*n* = 11), or having it covered by their health insurance (*n* = 7) (missing data: *n* = 1).

**Table 2:** Descriptive Statistics of Use of Complementary Medicine in the past 6 months (*N* = 123)

Complementary medicine	*n* (%)^1^
None	87 (70.7)
**Alternative medical system**	
Acupuncture	2 (1.6)
Homeopathy	4 (3.3)
**Biologically-based therapies**	
Nutritional supplements	8 (6.5)
Herbal medicine (phytotherapy)	2 (1.6)
Aromatherapy	2 (1.6)
**Mind-body-based methods**	
Meditation	9 (7.3)
Animal-assisted therapy	1 (0.8)
Music therapy	8 (6.5)
Hypnosis	0 (0)
Art therapy	3 (2.4)
Yoga	3 (2.4)
Sophrology^2^	1 (0.8)
**Manipulative and body-based methods**	
Therapeutic massage	10 (8.1)
chiropractor, osteopath	5 (4.1)
**Energy therapies**	
Reiki	0 (0)
Tai-Chi, Qi-Gong	0 (0)
Healer, secret	3 (2.4)

Note. ^1^Number and percentage of participants reporting having used the complementary medicine service in the past 6 months at least once. ^2^Refered to as a relaxation method including physical and mental exercise

### Interest in CM

Table 3 displays participants’ reported interest in CM and Table 4 presents the same results stratified by nationality (European or Swiss vs. non-European). In the whole sample, the top 5 most popular CM included chiropractor and/or osteopathy, therapeutic massage, nutritional supplements, music therapy and acupuncture. These were among the five most endorsed CM approaches across non-European and European participants.

**Table 3:** Descriptive Statistics of Interest in Complementary Medicine in the Whole Sample (*N* = 123)

Complementary medicine	Very interested or interested^1^	Very interested^1^	Interested^1^	Indifferent^1^	Not interested^1^	Not interested at all^1^	Missing
**Alternative medical system**							
Acupuncture	**49.2**	26.3	22.9	7.6	4.2	39	5
Homeopathy	36.8	15.4	21.4	12	6	45.3	6
**Biologically-based therapies**							
Nutritional supplements	**57.2**	28.6	28.6	6.7	3.4	32.8	4
Herbal medicine (phytotherapy)	46.6	25.8	20.8	10.8	6.7	35.8	3
Aromatherapy	45.4	18.5	26.9	8.4	6.7	39.5	4
**Mind-body-based methods**							
Meditation	43.2	20.3	22.9	9.3	9.3	38.1	5
Animal-assisted therapy	31.1	16	15.1	10.9	5.9	52.1	4
Music therapy	**50.9**	26.7	24.2	8.3	3.3	37.5	3
Hypnosis	26.1	16	10.1	13.4	8.4	52.1	4
Art therapy	41.1	23.5	17.6	10.1	5	43.7	4
Yoga	34.7	12.7	22	6.8	6.8	51.7	5
Sophrology	35.6	13.9	21.7	11.3	6.1	47	8
**Manipulative and body-based methods**							
Therapeutic massage	**59.2**	40	19.2	6.7	4.2	30	3
Chiropractor, osteopath	**61.6**	38.5	23.1	4.3	2.6	31.6	6
**Energy therapies**							
Reiki	25.4	12.7	12.7	11	7.3	55.9	5
Tai-Chi, Q-Gong	26.5	12.8	13.7	11.1	7.7	54.7	6
Healer, secret	37.8	21.8	16	8.4	2.5	48.7	4

Note. ^1^Percentages. Numbers in bold stand for the top 5 most popular approaches

**Table 4:** Descriptive Statistics of Interest in Complementary Medicine by Nationality

Complementary medicine		European and Swiss participants (*n* = 72)							Non-European participants (*n* = 51)					
	VI^1^ or V^1^	VI^1^	I^1^	Ind^1^	NI^1^	NI at all^1^	Missing	VI^1^ or V^1^	VI^1^	I^1^	Ind^1^	NI^1^	NI at all^1^	Missing
**Alternative medical system**														
Acupuncture	**43.5**	26.1	17.4	8.7	4.3	43.5	3	**57.1**	26.5	30.6	6.1	4.1	32.7	2
Homeopathy	29.4	11.8	17.6	14.7	8.8	47.1	4	46.9	20.4	26.5	8.2	2	42.9	2
**Biologically-based therapies**														
Nutritional supplements	**53.5**	29.6	23.9	5.6	2.8	38	1	**62.5**	27.1	35.4	8.3	4.2	25	3
Herbal medicine (phytotherapy)	43.7	26.8	16.9	11.3	5.6	39.4	1	51	24.5	26.5	10.2	8.2	30.6	2
Aromatherapy	37.2	14.3	22.9	8.8	8.8	45.7	2	**57.2**	24.5	32.7	8.2	4.1	30.6	2
**Mind-body-based methods**														
Meditation	35.2	14.1	21.1	12.7	9.9	42.3	1	55.3	29.8	25.5	4.3	8.5	31.9	4
Animal-assisted therapy	29.6	16.9	12.7	14.1	7	49.3	1	33.4	14.6	18.8	6.3	4.2	56.3	3
Music therapy	**45**	23.9	21.1	9.9	5.6	39.4	1	59.2	30.6	28.6	6.1	0	34.7	2
Hypnosis	24	15.5	8.5	14.1	8.5	53.5	1	29.2	16.7	12.5	12.5	8.3	50	3
Art therapy	35.2	14.1	21.1	12.7	5.6	46.5	1	50	37.5	12.5	6.3	4.2	39.6	3
Yoga	30.4	10.1	20.3	8.7	7.2	53.6	3	40.8	16.3	24.5	4.1	6.1	49	2
Sophrology	29.8	13.4	16.4	10.4	7.6	52.2	5	43.8	14.6	29.2	12.5	4.2	39.6	3
**Manipulative and body-based methods**														
Therapeutic massage	**50.7**	32.4	18.3	8.5	5.6	35.2	1	**71.6**	51	20.4	4.1	2	22.4	2
Chiropractor, osteopath	**58.8**	38.2	20.6	5.9	2.9	32.4	4	**65.3**	38.8	26.5	2	2	30.6	2
**Energy therapies**														
Reiki	22.6	8.5	14.1	14.1	8.5	54.9	1	29.7	19.1	10.6	6.4	6.4	57.4	4
Tai-Chi, Q-Gong	26.5	14.7	11.8	10.3	7.4	55.9	4	26.5	10.2	16.3	12.2	8.2	53.1	2
Healer, secret	38.6	24.3	14.3	10	4.3	47.1	2	36.8	18.4	18.4	6.1	0	57.1	2

Note. VI = very interested; I = interested; Ind = indifferent; NI = not interested; NI at all = not interested at all; ^1^Percentages

## Qualitative results

A subset of those who participated in the survey (*n* = 18; 72% men) and professionals (*n* = 14; 64.3% women; emergency night shelter watcher = 6; social workers = 3; nurses = 2; CM practitioners = 2; peer helper with lived experience of homelessness = 1) completed semi-structured interviews. Four topic areas emerged from the analysis: knowledge and experience of CM among PEH; interests in receiving CM and perceived usefulness for PEH; recommendations to engage and retain PEH in a CM program; and considerations for developing a CM program offering for PEH: is this really the priority? The topics arising through analysis are summarized in Table 5.

**Table 5:** Summary of Qualitative Findings Highlighting the Main Topics Describing PEH Perceptions of CM

**Topic 1: Knowledge and Experience of CM among PEH** Most participants reported at least one previous experience of CM, including herbal medicine, therapeutic massage, osteopathy, chiropractic, yoga, and meditation.	
**Topic 2: Interests in Receiving CM and Perceived Usefulness** **Categories** • **Relieving Pain and Helping Face Social Deprivation with Osteopathy, Therapeutic Massages and Acupuncture:** corporal approaches perceived as responding to a concrete pain for people living in difficult conditions also benefiting affects and well-being. • **Music Therapy:** The Power of Music to Enhance Well-Being, Relaxation and Socialization: Music (therapy) was seen as important in the daily lives of PEH and promoting self-expression. • **Meditation and Yoga can Decrease Stress Related to Homelessness:** these approaches were perceived as having a positive impact on relaxation and clearing the mind for people facing numerous problems and stresses. • **Animal-assisted Therapy to Help Face Social Isolation and Stigmatization and Improve Self-confidence and Self-esteem:** this therapy was perceived as an opportunity to share affection (with dogs or horses) and ultimately promote social capacities. • **Herbal Medicine to Avoid Conventional Medicines and Nutritional Supplements to Compensate for Irregular Meals:** these approaches were seen as useful to help face chronic pain and nutritional deficiencies. • **Not interested in CM Approaches or Being Unsure: **a few participants expressed reluctance to CM approaches due to never having used or not knowing about CM. • **Individual or In-group Sessions:** most participants were favorable to a group setting, encouraging experience sharing and engagement.	
**Topic 3: Recommendations to Engage and Retain PEH in a CM Program** **Categories** • **Make it Equitable and Accessible:** make it access free and without criteria such as demographic factors and implement the program in the low-barrier existing structures during the weekend. • **Earning the trust of PEH:** collaborate with existing low-barrier structures, provide information about the program, guarantee flexibility, and create a warm and welcoming environment.	
**Topic 4: Considerations for Developing a CM Program Offering for PEH: is this Really the Priority?** **Categories** • **Improving the Access to Conventional Medicine and Other Para-Medical Services First**: for a minority of participants, it is more urgent to facilitate access to conventional medicine, mental health care and podiatry. • **Fulfilling Basic Social Needs First**: PEH face urgent social needs (eat, have a secure and stable place to sleep, find a job/occupation) which must be prioritized according to several participants.	

### Knowledge and experience of CM among PEH

When asked about their use of CM, most PEH reported at least one previous experience. These experiences were commonly related to childhood memories in participants’ country of origin, with herbal medicine being among the most often-cited approaches. One participant stated for instance:

I remember, when I was young, in Africa, my grandmother had natural solutions, plants that we used to take in the morning (…). According to the belief, it helped fight against malaria, intestinal worms. Listen, it was at my young age, so I don’t know, but there was a strong belief in that (PEH, man).

A few participants recalled using other forms of CM, including therapeutic massage, osteopathy, and chiropractic. For instance, a participant (PEH, woman) reflected on the time she used to work as a housekeeper and suffered from backache: “I called for massage (…). I suffered from backache because of 8 hours of housekeeping per day (…). It helped me 100%. Massage and osteopathy, both. Because it’s nervous fatigue when you do this kind of job.” Other participants reported having practiced yoga and meditation. For instance, reflecting on when she took care of her young child while also dealing with a chronic disease, a participant shared a positive experience of meditation:

At that time, I remember I did a lot of meditation, well, even though I also had to take care of my son and so on (PEH, woman).

Most participants recounted positive feelings towards prior experiences with yoga and meditation. However, one participant’s (PEH, woman) testimony was more nuanced, sharing that “yoga was a resource that [she] had developed,” yet warning “if one is traumatized, one really must be careful with these things,” remembering that “meditation didn’t feel good once or twice.” Finally, around one quarter of the participants mentioned that they had never used any form of CM in the past, most often because they were not given the opportunity.

### Interests in receiving CM and perceived usefulness

Most participants expressed interest in using CM, which was perceived as a potential means to help mitigate some health and social difficulties and ultimately improve health and well-being.

#### Relieving pain and helping face social deprivation with osteopathy, therapeutic massages and acupuncture

Many participants, both PEH and professionals perceived certain body-based CM therapies as useful to help relieve pain: “I feel like a corporal approach that responds to a physical concrete pain, it’s a good idea (professional, woman).” Reinforcing this idea, while evoking the rough conditions related to homelessness, another professional (man) mentioned that osteopathy might be a good fit: ”In terms of general well-being, I think there’s a lot of work to be done: providing these people with an osteopath they can go to. People who sleep on the sidewalks would really benefit from this kind of treatment.” Relatedly, while evoking his practice in a low-threshold medical center, a professional explained:

Often people [PEH] consult us for somatic problems, such as back pain, headaches, or tension in the neck. And when these people can already get massages, more of the relaxation type, or even acupuncture, alternatives to just prescribing medication, they’re relieved in fact (professional, man).

Consistently, PEH commonly expressed interest in receiving osteopathy to help mitigate pain. For instance, speaking about osteopathy, a participant (PEH, man) shared: “It hurts the way I am now, yes, that’s something [osteopathy] I’d be interested in.” He went on to explain that such an approach would enable him “not to wait until it becomes serious and would bring relief.”

Another popular body-based CM was therapeutic massage. For instance, a participant (PEH, woman) disclosed: “I like massages. It’s total bliss when you get a massage, you feel light!” Consistently, while reflecting on the difficult conditions of homelessness, several professionals mentioned that massages represent a way to help PEH relax and improve well-being:

Massages, I think that their body takes a lot of things. It’s just being on the street that makes the body … it picks up, so to speak. So, I think that (…), if they can also relax and, accept the thing, well, I think that would be ideal (professional, woman).

Well, at [low barrier healthcare organization] we offer them massages. (…) It helps them rediscover (positive) sensations with their body. That works very well, we often get good feedback from them (professional, woman).

Relatedly, while recognizing the therapeutic limits of massage, a few participants noticed the complementarity of other approaches, such as acupuncture. A participant (PEH, man) disclosed for instance:

Therapeutic massage can be useful, if you’ve got body aches and pains and all that, if it’s well done, it can tone you up, yeah. But it can’t cure. Acupuncture can because it touches the nervous system.

Considering the social deprivation faced by many PEH, a few participants reflected on the potential affective benefit of physical touch during an osteopathy or massage session:

They don’t have any, um… physical contact. And it’s part of being human to be touched, to have contact. When it’s not there, there’s a kind of… of dryness inside. (…) Touching them, relaxing them, being kind, (…) it does them a lot of good to have this thing that’s a bit fair. There’s nothing at stake, it just feels good. I think that’s really important (professional, woman).

#### Music therapy: the power of music to enhance well-being, relaxation and socialization

Although few participants had prior experience with music therapy, when asked about their interest in it, many answered positively while disclosing the importance of music in their daily life:

You’re living in a country that’s good but you’re still afraid… It sticks to you like a perfume of stress daily so you cannot have the joy to appreciate all things (…) but when I find myself playing the guitar, piano, singing… It’s good. (…) After a while, sometimes I forget my worries, sometimes I’ve had suicidal thoughts… without the guitar maybe I wouldn’t be here anymore (PEH, man).

Another participant reported:

Music does good to you and others. Music is what I love; I’ve got a radio with loudspeakers and then I put on the crazy music and people who know it, give me a little money too…. It’s heartwarming. The police came once, I thought he was going to ask me to stop it. He said “No, you mustn’t stop, it’s good for the heart, it’s good for the mood, leave the music on.” So even the policeman appreciated it (PEH, man).

Corroborating these reports, professionals commonly reflected on the importance of music in daily life for PEH and perceived that music therapy could help address some of this population’s needs:

Why not music therapy? It’s true that we have quite a few people who have nothing on a day-to-day basis, so we try to work on what makes them feel good, little things to help them. So sometimes it’s playing an instrument, but sometimes it’s just basic stuff, like listening to music or singing; and a lot of people do that. (…) It’s true that music is something that helps them. (professional, female).

According to participants, PEH might benefit from music therapy in several ways, including self-expression and being able to use a learned skill. Some of them outlined the potential socialization effect of this approach. The fact that it can be provided in groups was perceived positively, so was the possibility to develop a shared project out of the groups, such “as a mini show that could bring them together” (professional, female). Next, both PEH and professionals perceived that music therapy might lead to “relaxation” and “soothing.” For instance, a participant (PEH, man) outlined the relaxing effects of nature sounds: “music therapy, yes, such as water music; it is relaxing; it is very good, you know when the water flows it really gives spirit tranquility.”

#### Meditation and yoga can decrease stress related to homelessness

Participants frequently considered that other mind-body based CM favoring relaxation may fit the target population’s needs. For instance, perceiving that “many [PEH] are stressed out, tense and keep everything inside,” a participant (professional, woman) noticed “another thing that might address their needs is having relaxation sessions,” and added knowing that “many would be interested.” While reflecting on the condition of homelessness, another professional reported:

When you’re on the street and you’ve got a lot of problems and everything seems, insurmountable (…). So, I’m thinking of stuff that helps clear your head, like meditation. Because I feel it’s a lot like that, where it doesn’t stop, and there’s no possibility of clearing the mind or just letting things go (man).

Echoing these assumptions, PEH commonly showed interest in yoga and in meditation. For instance, a PEH stated:

Yoga, yes, I’m interested in that (…). Yoga, meditation, all that, it’s good. I did maybe 2 or 1 weeks like that, but then I didn’t do it again. I’d like to, if I had the opportunity to do it again, to sign up somewhere, I’d like to (man).

When asked why he was interested in yoga, he stated:

It’s a question of refocusing on oneself, (…) regaining the self-confidence that we’ve sometimes lost… This calming, unloading everything that’s negative, regaining the good energy (…). I know that yoga, meditation, and music are things that make me feel good again… And I really miss that!

#### Animal-assisted therapy to help face social isolation and stigmatization and improve self-confidence and self-esteem

Around one third of participants expressed interest in animal-assisted therapy, involving dogs and/or horses. A participant (PEH) shared for instance: “For me, it would be animal therapy; (…); it would be such a pleasure to know them [animals] and get their affection.” Similarly, another participant (PEH, man) mentioned he would be interested in therapy with dogs and horses, explaining:

I love animals, especially dogs, horses. It can be useful on a psychological level. For people who are alone, they give all their love they could give elsewhere, they give it to the animal. And sometimes if they feel it [love], the animal feels it, too.

Corroborating these reports, several professionals perceived that animal-assisted therapy might interest some PEH, similarly outlining the affection sharing as the main therapeutic mechanism: “Animals, it could work well; there’s the affection many people lack” (professional, woman). In fact, considering the social isolation and stigmatization typically experienced by PEH, animal-assisted therapy was sometimes perceived as potentially bolstering self-confidence and self-esteem:

I think that we are at risk of having a drop in self-confidence and self-esteem and that animal therapy could help (…); we’re a bit left out. (…) We don’t really take part in the usual social activities, we don’t necessarily have contact with friends anymore because for them it’s delicate to be with someone who’s homeless. So, in terms of trust, I find that being in contact with animals allows you to… to keep a benevolent view of yourself. Because you immediately regain self-confidence when you see that the dog trusts you and that you can create a bond. (…) It allows us to anticipate our future relationship with people, with society. It’s very important to reconnect, perhaps via animals, with this confidence that we’ve lost, this self-esteem that we’ve lost, the… animal doesn’t judge us (PEH, woman).

#### Herbal medicine to avoid conventional medicines and nutritional supplements to compensate for irregular meals

Although less common in the qualitative inquiry, a few participants showed interest in biologically based therapies, such as herbal medicine and nutritional supplements. For instance, participant (PEH, man) disclosed “believing in herbal medicine” and added: “I’m a curious person, I like to find out about which herbs can cure a headache and choose the herb myself. I would buy it, and I make the infusion myself.” Echoing this, another participant (professional, man) reflected on the potential usefulness of herbal medicine to help face chronic pain:

I’ve seen a lot of people [PEH] who had enormous chronic pain (…). I was often very surprised at how medicated they were, and how it didn’t change a thing. I found that alternative herbal medicines, whether it’s THC, CBD, or other things, could be very interesting for chronic pain.

Regarding nutritional supplements, a few participants perceived that they may be useful to compensate for irregular meals and associated nutritional deficiencies:

I’d stress the importance of food supplements in terms of the vitamins you need… We should be able to keep ourselves in shape, knowing that we don’t always have three meals a day…. It might be better to… at least try to supplement…. My concern is that we don’t wither away (PEH, man).

#### Not interested in CM approaches or being unsure

A minority of participants reported being unsure or not being interested in CM, commonly because they had never used any of them or did not know them. For instance, while talking about CM, a participant (PEH, man) said: “No, I’m not interested in that thing. I don’t know it, and I don’t really want to know it,” whereas another explained:

I haven’t… often been in contact with CM. So, it’s really hard for me to say. Homeopathy, phytotherapy, and sophrology. Well, I’ve heard of it, but I don’t know anything about it. (…) Not having exact knowledge of how it works, I find it hard to make choices because I’m stuck in what I know, in what I have used. But otherwise, why not (PEH, man).

Finally, some of the participants shared reservations about certain practitioners, such as healers, questioning their seriousness and urging caution. A participant (PEH, woman) disclosed for instance: “Not interested in the healer. I find that a bit strange and it doesn’t seem very serious to me.”

#### Individual or in-group sessions

Irrespective of the CM approach, participants gave their opinion on providing the offering in individual versus in-group settings. A minority of participants were more positive about sessions delivered in individual settings, evoking that in-group sessions would not be feasible because “there’s not much affinity in this population, there are fights and insults” (PEH, man) or individual preferences:

I wouldn’t like to do group things. I really need to be one on one… with a closed door. I don’t like being exposed to other people discussing my problems, because it’s vulnerable and it’s political. It’s not for everyone either, and I feel exposed in the public already against my will a lot (PEH, woman).

In contrast, other participants preferred the in-group setting, which was perceived as enabling “gathering people” and “sharing experiences.” Likewise, all professionals favored group settings whenever possible, often evoking that it might help users to be more engaged:

I imagine something in a group. Because I think it also motivates [them]. There could be emulation too; to see that they’re not the only ones (…), because there might also be the apprehension of trying something new, they’re not too familiar with and maybe it’s easier if there’s more than one of them. (…) Yeah, maybe it brings them some reassurance (professional, man).

### Recommendations to engage and retain PEH in a CM program

Beyond favoring the in-group session, participants made recommendations to engage and retain PEH in a hypothetical future CM program.

#### Make it equitable and accessible

First, participants frequently mentioned that the offer should be accessible to everyone. Reflecting on the fact that in the Canton of Vaud, access to night-shelters typically vary depending on gender, residency permits, age or working situation, a participant (professional, woman) shared: “It should be conditional on nothing; it should be an unconditional welcome.” Relatedly, participants commonly made several recommendations to enhance the program’s accessibility, such as making it totally free and offering it in users’ languages. Likewise, participants commonly recommended providing the program in outreach settings, such as those easily accessible with public transport or in city centers. In the same vein, participants advised that the intervention should be provided in existing low-barrier structures, such as day-time drop-in centers or emergency night shelters.

According to participants, enhancing the accessibility would also depend on the timing of the program. Reflecting on the fact that many PEH have jobs or occupations during the weekdays and that in Switzerland, most services, shops, and community programs are closed on Sundays, participants commonly advised to provide this offer during the weekend:

The time when you’re bound to attract the most people is when the workers have the day off, and the others have nothing to do. (…) The migrant worker works from Monday to Friday on a construction site, so he’s not going to take Wednesday afternoon off to go and take care of himself. (…) Reaching out to others is especially important on Sundays. Because the rest of the week there’s an interest in hanging around on the street for money and begging (professional, male).

#### Earning the trust of PEH

Many participants insisted on the importance of following ground rules to earn trust and engage PEH in a CM program. First, professionals consistently recommended to collaborate with existing low-barrier structures’ professionals who already have a relationship with PEH to promote the CM program and earn their trust:

It’s important to share with us, who are out in the field and know them [PEH] in a different way. Because some of them will come to us and say: “what’s all this?” And we might be able to approach them differently (professional, man).

Next, echoing the previously described perceptions that interest in engaging in a CM program might be dampened by the fact that PEH might not be familiar with it, several participants advised to provide information and make sure the target population understands what it entails and how they could benefit from it. Professionals often recommended to provide information in existing low-barrier organizations and to organize open-house days to have people discover the program. Relatedly, participants often stated that word-of-mouth might work well to have the offer known and to engage the target population. While advising to set up the offer in a low-barrier organization, a participant (professional, woman) added: “Actually really do… don’t just propose and wait for people to come but do something that attracts attention. And as a result, people who are curious will get involved, and that’ll lead to other people.” Similarly, other participants insisted on the importance of consistently showing up and being patient:

Above all it’s about being present, for me that’s what can make the thing work (…). Being present changes everything, to do it in person (…). They’re not going to go, no, they’re going to watch from afar and then little by little they’re going to get involved; (…) once they’re on site, [they will see that] there’s room for them, the setting is pleasant, it would be very useful and then they could get involved and see that you’re there for them (professional, male).

Relatedly, some participants insisted on humanistic skills that the practitioner providing the CM program should have. According to a participant (professional, female) for instance, “it would take someone who has a real interest in providing this kind of service in emergency shelters, someone who would have a great ease in creating relationships.” Similarly, another participant (PEH, male) noticed: “You need the right person, having a supportive relationship; because it’s the relationship, with the practitioner, who shares the thing with you. So, it can work.”

Finally, a few participants suggested using several strategies to enhance engagement and retention over time, such as being very flexible (regarding CM session attendance for instance). Related to this idea, some participants recommended to offer a warm and friendly setting to gain loyalty among users and foster a sense of community over time. According to a participant (professional, woman), this could be achieved by offering a gift object “maybe a T-Shirt or a water bottle; something that makes the person feel integrated” and by proposing every time small moments to share a snack: “I think that it’s also [important that there are opportunities] to meet - not just to do the activity, but that there is a possibility after, just to share a snack. That works well.”

### Considerations for developing a CM program offering for PEH: is this really the priority?

#### Improving the access to conventional medicine and other para-medical services first

Despite the uniformly high level of interest in CM, a minority of participants questioned its relevance, noting that there are more urgent conventional medical needs among PEH which should be addressed first. A few participants perceived that improving access to conventional medicine would be more important than offering CM:

Basic things, for me it’s more important than doing the alternative. More access to medication, for people who don’t have any insurance or any means. (…) I only have a few coins (…) so it’s not much to buy medication. (…) Having access to medication when you need it would be my priority, having access to your own doctor if you’re sick. Things like that are… much more important to me than alternative things (PEH, woman).

Similarly, another participant voiced:

I say people who are homeless still have the right to have care, doctors, we’re not talking about massages or complementary stuff, but at least the basics (…). Every person with a home or without a home, has the right to see a doctor, with a permit or without a permit… a sick person, he must be helped, he must have hope. (…) When you don’t have your health, well, you can’t do anything (PEH, woman).

Furthermore, a few PEH stated that they would seek mental health care providers before wishing for CM programming. For instance, when asked about which kind of healthcare professionals (including CM practitioners) he would like to meet, a participant (PEH, man) answered: “One day I’d like to have a psychologist, if that’s possible. Because it’s hard.” Echoing these reports, several professionals spontaneously outlined the importance of mental-health problems commonly faced in this population and the critical need to enable access to mental health care. For instance, a participant (professional, man) reflected on the impact of homelessness on mental health: “As soon as a person has lost their home and has to sleep outside, (…) what struck me the most was the psychological impact it can have, and the speed at which it can go.” While witnessing severe mental health problems, several professionals denounced the lack of access to mental health care in this population:

I find that there are quite a few people who have psychiatric problems. (…). We really have people who are not well, and we don’t know where to send them (…). If they had the chance to contact health services, we might have been able to get them back before things got too bad in terms of their health (professional, woman).

Finally, although it did not spontaneously emerge among PEH, half of the professionals interviewed perceived podiatry services as a critical need. For instance, when asked about which kind of treatment offers should be developed for the target population, some participants answered:

A treatment that could be a good thing, (…) would be more a form of podiatry because I know that physically they suffer a lot from their feet. And I’d heard that it could also do a lot of good in a general way (professional, woman).

#### Fulfilling basic social needs first

Several participants highlighted that PEH typically face urgent social needs which must be prioritized. These may include eating, finding a job/occupation, and a secure and stable place to stay. For several professionals, engaging with CM might be perceived as secondary to these more urgent social needs:

Well, if one reflects on Maslow’s pyramid… People are at that level of need, they need a roof over their heads, they need to eat, maybe they need a substance, and that’s the priority. And when we come up with offers for, personal development, we’re right at the top of the pyramid (professional, woman).

Echoing these perceptions, several PEH emphasized the priority of fulfilling basic social needs first. For instance, when asked about his interest in CM, a participant (PEH, man) answered: “I worry about where to sleep, where to eat, about work, it’s already quite a lot in my head, I don’t have time to be well… I get that without health there’s no work, but one must survive first.” Likewise other participants disclosed their urgent needs:

If I see some work to do, cleaning or anything, to work to find money for my children… some cash to buy food, I will be peaceful in life. I will be thinking normal like a human being (…). What can help me like here in my situation now, something to have a little better life. Like have a place to stay, and doing some work (PEH, man).

Close to these ideas, professionals frequently reported a critical need to create dedicated daytime low-barrier centers, offering a place to stay and occupy oneself during the days, wherein the type of programming (i.e., CM or something else) was perceived as secondary:

It’s important to fill in the gaps during the day, it doesn’t really matter what you do, because when we [night emergency shelters] close, people find themselves out on the street, and then the days are long; they have nothing. Really in the street, with nothing to do, no place to spend time and then with the weather, it’s cold, that’s the thing, it kills (professional, man).

## Discussion

This study aimed to describe the perceptions, experiences, and interests regarding CM among PEH in the French-speaking part of Switzerland. Main quantitative findings showed that, despite high levels of interest in CM, less than one in three participants reported having used CM at least once in the previous 6 months. The five most popular CM approaches were osteopathy and/or chiropractic, therapeutic massage, nutritional supplements, music therapy and acupuncture. High interest in CM was confirmed by qualitative findings, although a minority questioned its relevance, arguing that more urgent social and conventional medical needs must be fulfilled first.

### CM might meet some specific needs and help address some health and social issues frequently encountered among PEH

Our findings, supportive of CM among PEH in French-speaking Switzerland, are aligned with international emerging results showing that CM are typically well received among PEH and that they might meet some of this population’s specific needs and expectations [28, 29, 33, 43, 44]. Participants perceived certain CM modalities as well positioned to help address several health issues frequently encountered among PEH, such as pain or psychological stress. These findings are reinforced by the results of the sister study conducted in the same sample confirming low levels of health-related quality of life, high levels of psychological distress and documenting that the most common health issues were musculoskeletal, dental, and psychiatric [40]. In line with participants’ perceived usefulness of CM to improve their health issues, CM are now well recognized as first-line treatment for certain health issues, such as chronic pain [45], although access might be limited due to costs [30, 31]. In fact, emerging evidence from the international literature has shown promising effects of acupuncture, mindfulness-based meditation, yoga, or nutritional counseling in improving both physical and psychological well-being among PEH [33, 36, 43, 44]. Therefore, our findings related to both interests and health-related needs suggest that actions improving access to CM are timely and might represent a promising means to help improve health equity in this population.

Furthermore, other qualitative findings revealed that certain CM, including animal-assisted therapy and music therapy, were perceived as well positioned to help counter social deprivation typically faced by PEH. As an extreme manifestation of social exclusion, homelessness often leads to a loss of social and affective links, resulting in loneliness [46, 47] and ultimately increased risks of depression [48]. More than 30% of the participants reported being interested in receiving animal-assisted therapy. Although not focused on animal therapy per se, past research showed that PEH typically experience strong bonds with their animal companions, compensating for reduced social links thereby buffering the negative impact of loneliness and ultimately resulting in improved emotional and physical well-being [49, 50]. Likewise, other qualitative findings highlighted that music therapy was considered as another well-suited CM modality to help those facing social deprivation. Participants outlined the potential socialization effect of music therapy, through group-session delivery mode and shared music project development. In fact, there is preliminary evidence that music therapy may provide benefits to PEH, such as engaging in meaningful connection and support [35]. For instance, two case studies conducted among men experiencing homelessness in the USA concluded that music therapy was helpful to counter feelings of isolation and meet socialization and belonging needs [51, 52].

Hence, considering that PEH are known to be at higher risk to suffer from chronic pain, psychological stress [53], the findings of this study coupled with previous research indicate that integrating CM (e.g., osteopathy, acupuncture, meditation, yoga, music therapy) [29, 32, 33, 35, 36] in healthcare might be well positioned to contribute to addressing the complex needs of this underserved population. Further efforts are needed to implement and evaluate such integration effectiveness and efficacy on physical and psychological well-being among PEH.

### Actions are needed to remove barriers and improve access to CM for PEH

Quantitative findings documented that around 29% of participants had used at least one CM modality in the past 6 months (e.g., therapeutic massage), which is consistent with the average use of CM in the general population in Switzerland [54]. These apparently counterintuitive findings may be related to the fact that a health-community center in the main city of the Canton of Vaud provides PEH with very low-barrier CM services (e.g., osteopathy, therapeutic massages). Importantly, our findings revealed higher levels of interest than actual use of CM. The multiple complex health problems typically encountered by PEH might drive this high interest in CM. Consistent with our findings, previous research conducted in the general population of Switzerland showed higher use of CM among people with a poor self-perceived health status (i.e., 40% use in the last year) [55]. In fact, quantitative findings revealed that CM needs and expectations remained largely unmet in our sample. For instance, more than 62% of the participants disclosed being interested in receiving osteopathy or chiropractic treatment, whereas only 4% reported having used this approach in the last 6 months, suggesting a lack of access to a service that is desirable.

Given that there is a current drive to improve health equity for PEH in Switzerland, taking action to improve the accessibility of CM approaches might contribute to reducing some important disparities. Qualitative findings highlighted strategies that might be useful to counter practical barriers, including providing equitable and accessible (i.e., free) delivery of CM embedded in shelters and day drop-in centers. Other proposed strategies aimed at earning the trust of PEH might be useful to drive engagement. Participants recommended working with professionals who know the target population; to clearly describe the CM program offering; to be humanistic and offer a warm and friendly setting to gain loyalty among users and develop a sense of community over time. These recommendations are consistent with previous considerations made in the field [24, 38, 56].

As researchers develop, implement, and evaluate novel CM offerings, it is important to do so in conjunction with those who have lived experience, in line with the approach of Community-Based Participatory Research (CBPR). CBPR is an increasingly used research paradigm combining knowledge and actions to improve health among communities and ultimately improve health equity [57, 58]. Using CBPR may be well positioned to enable the proposed strategies because it offers a flexible and inclusive framework aimed at equitably involving community members, researchers, and stakeholders in the whole research process. Most important, there is increasing evidence of this paradigm’s relevance and effectiveness in improving health in socially disadvantaged groups, such as PEH [59, 60]. Hence, future research aimed at implementing a CM program for PEH should use this approach to ensure following the strategies highlighted in our qualitative findings and ultimately ending up with an equitable and accessible offer.

### Putting it all together: towards equitable integrative medicine for PEH?

Despite high levels of interest in CM, findings revealed that other medical (e.g., improving access to conventional care and mental health treatment) and social (e.g., ensuring food, improving access to housing and/or day-in drop-in centers) needs were perceived as more urgent. Taken together with the overall high levels of interest in CM these findings suggest that integrative medicine may represent a suitable contribution to aid PEH manage their competing bio-psycho-socio needs. Echoing our findings highlighting the priority to fulfill basic social needs, such integrative medicine programs tailored to PEH should incorporate social services. The latter should aim to address basic social problems that directly affect their health. Likewise, consistent with our findings identifying strategies to remove practical and emotional barriers to a CM program, such integrative medical program should ensure equitable access in outreach settings and be co-developed together with the community of people with relevant lived experience and the involved stakeholders. To build upon this, prioritizing the activation of patients’ individual resources (motivational dispositions and abilities) and not focusing solely on their needs in the therapeutic process could foster their empowerment and engagement in therapies included in integrative medicine. Our results highlighted positive childhood experiences, their interests, and expectations as resources for participants, as well as their recommendations on how to engage PEH in the program. This could have a beneficial impact on the therapeutic relationship and on the PEH’s self-esteem [61]. To illustrate this concept, a study assessing support groups for PEH with alcohol problems showed a significant association between the negative perception of therapy by PEH and lower motivation and treatment attendance [62]. Future research aimed to co-develop implement and evaluate equitable and outreach integrative medicine programs incorporating social services may benefit PEH in Switzerland. These research efforts should be done considering PEH CM prior experience, needs and interests.

### Limitations

The main limitation of this study relates to the specificity of the local social and health care system, limiting the transferability of our findings to other countries. Nevertheless, our findings are consistent with prior international research. Second, we cannot exclude sampling bias, as PEH and professionals who accepted to participate in the study may differ from those who did not, even though we used purposive sampling in the qualitative inquiry to mitigate this risk. Third, the quantitative study was comprised of a self-reported questionnaire, which can be subject to reporting bias. To limit this risk, the administration of the questionnaire was conducted by trained research staff with professional interpreters to ensure understanding when needed and involved validated questionnaires. Finally, triangulation of quantitative and qualitative data contributes to the trustworthiness of our findings. Using both methods enabled a more complete description of the topic than could have been reached using a single method.

## Conclusions

This quantitative and qualitative study aimed to explore the perceptions, experiences, and interest in CM among PEH in Switzerland. The main findings revealed high levels of interest in CM (e.g., osteopathy, therapeutic massage, music therapy) among PEH in the Canton of Vaud in Switzerland, who perceived that these approaches are well positioned to mitigate some negative consequences of homelessness. Taken together with findings highlighting the importance of fulfilling basic medical and social needs first, these results suggest that integrative medicine, (i.e., incorporating conventional and complementary medicine approaches and social services) would be a desirable proposition to aid PEH manage their competing bio-psycho-social needs. Using a community-based participatory paradigm to co-develop such a program might ensure that the program developed is equitable and meets peoples’ needs.

## Electronic Supplementary Material

Below is the link to the electronic supplementary material.

Supplementary Material 1

Supplementary Material 2

## Data Availability

No datasets were generated or analysed during the current study.

## Abbreviations

PEH: People Experiencing Homelessness
CM: Complementary Medicines
