# Supplementary material for: Complementary medicine among individuals experiencing homelessness in Switzerland: a quantitative and qualitative descriptive study
Source: BMC Complement Med Ther. 2025 May 6;25:166. doi: 10.1186/s12906-024-04727-4 (PMC12057206; doi:10.1186/s12906-024-04727-4)
Supplement: Supplementary file 1 — Supplementary Material 1 [file 12906_2024_4727_MOESM1_ESM.pdf]

Appendix 1: questionnaire

Research project on the health of people experiencing homelessness in the canton of Vaud and integrative Medicine.

Questionnaire phase 1 identification of CM needs and interests

Interest in complementary medicine approaches of homeless facility users

Partner institution (where the questionnaire is completed): ____

Date: ____/____/_________________

Research Assistant [initial]: _______________

Demographics

*The first questions concern your personal situation. The questionnaire is **confidential**. All information will **only be known** by the important members of **the research team.***

1. What is your gender?

- ☐ Man
- ☐ Woman
- ☐ Other

2. How old are you? ______________

3. How long have you been living in Switzerland? _______________

4. What is your nationality? [1 possible answer].

- ☐ Switzerland (1)→ *go to question 7*
- ☐ European (2) (specify country) : ______________
- ☐ Non-European (3) (specify country) : _________________________
- ☐ I do not know
- ☐ I do not wish to answer (999)→ *go to question 7*

5. What's the type of your residence permit? [1 answer possible]

- ☐ B (1)→ *go to question 7*
- ☐ C (2)→ *go to question 7*
- ☐ F (3)→ *go to question 7*
- ☐ G (4)→ *go to question 7*
- ☐ L (5)→ *go to question 7*
- ☐ N (6)→ *go to question 7*
- ☐ S (7)→ *go to question 7*
- ☐ B on grouping (8)→ *go to question 7*
- ☐ None (777)
- ☐ I don't know (888)
- ☐ I don't wish to answer (999)

6. What's your current situation? [1 possible answer]

- ☐ No application for asylum (1)
- ☐ Non-entry (2)
- ☐ Refusal of the asylum application (3)
- ☐ Dublin case (4)
- ☐ I don't know (888)
- ☐ I don't wish to answer (999)

7. **What's your highest level of education**? [1 answer possible]

- ◻ No training (schooling not completed) (1)
- ◻ Compulsory school (2)
- ◻ Higher secondary school diploma (high school, college) (3)
- ◻ General and vocational training (CFC) (4)
- ◻ Higher education (UAS, University) (5)
- ◻ I don't know (888)
- ◻ I don't wish to answer (999)

8. **Do you have health and accident insurance?** [1 answer possible]

- ◻ Yes (1) → If yes, is the insurance? : ☐ Swiss (2) ☐ foreign (3)
- ◻ No (4)
- ◻ I don't know (888)
- ◻ I don't wish to answer (999)

9. **How long have you been homeless?** _____________

10. **During the nights of the past 2 weeks, where did you sleep most often?** [1 possible answer]

- ◻ Homeless (on the street, shelters in public space, etc.) (1)
- ◻ In an emergency accommodation (Sleep-In, Marmotte, Lucarne, etc.) (2)
- ◻ In a hostel or shelter (e.g. EVAM centers) (3)
- ◻ At a relative's home (a friend or member of my family) (4)
- ◻ Non-conventional housing (mobile homes, camping, squats, temporary structures) (5)
- ◻ Individual housing (tenant or owner) (6)
- ◻ Prison institution (prisons) (7)
- ◻ Health institution (hospital or other) (8)
- ◻ I don't know (888)
- ◻ I don't wish to answer (999)

Use and interest in complementary medicine approaches

1. In the past 6 months, what type(s) of complementary medicine(s) have you used? [Multiple answers possible; explain each approach using the instructions received during the training]

- ☐ None (0)
- ☐ Acupuncture (1)
- ☐ Therapeutic massage (2)
- ☐ Reiki (3)
- ☐ Meditation (4)
- ☐ Animal-assisted therapy (5)
- ☐ Food (nutritional) supplements (6)
- ☐ Music therapy (7)
- ☐ Hypnosis (8)
- ☐ Art therapy (9)
- ☐ Phytotherapy (Herbal medicine) (10)
- ☐ Chiropractor, osteopathy (11)
- ☐ Tai-chi, Qi gong (12)
- ☐ Yoga (13)
- ☐ Homeopathy (14)
- ☐ Aromatherapy (15)
- ☐ Sophrology (Relaxation therapy) (16)
- ☐ Healer/Secret (17)
- ☐ Other(s) (18): ________________________________

2. If you have used one or more of these complementary medicines, how were they paid?

- ☐ Basic insurance (1)
- ☐ Additional insurance (2)
- ☐ Cash payment (3)
- ☐ Free treatment (4)
- ☐ Other (888): specify______________________

The final questions ask about your interest in (re)pursuing a ***complementary medicine therapy****. For each type of complementary medicine listed below, please indicate how much you would like to be treated with that practice.* [Explain each approach using the instructions received during the training]

3.How much would you like to be treated with	**Very interested**	**Interested**	**Indifferent**	**Not very interested**	**No interest**
**Acupuncture?**	(1) ☐	(2) ☐	(3) ☐	(4) ☐	(5) ☐
**Therapeutic massage?**	(1) ☐	(2) ☐	(3) ☐	(4) ☐	(5) ☐
**Reiki?**	(1) ☐	(2) ☒	(3) ☐	(4) ☐	(5) ☐
**Meditation?**	(1) ☐	(2) ☐	(3) ☐	(4) ☐	(5) ☐
**Animal-assisted therapy?**	(1) ☐	(2) ☐	(3) ☐	(4) ☐	(5) ☐
**Use of nutritional supplements?**	(1) ☐	(2) ☐	(3) ☐	(4) ☐	(5) ☐
**Music therapy?**	(1) ☐	(2) ☐	(3) ☐	(4) ☐	(5) ☐
**Hypnosis?**	(1) ☐	(2) ☐	(3) ☐	(4) ☐	(5) ☐
**Art therapy?**	(1) ☐	(2) ☐	(3) ☐	(4) ☐	(5) ☐

3.How much would you like to be treated with	Very interested	Interested	Indifferent	Not very interested	No interest
**Herbal medicine?**	(1) ☐	(2) ☐	(3) ☐	(4) ☐	(5) ☐
**Chiropractor, osteopathy ?**	(1) ☐	(2) ☐	(3) ☐	(4) ☐	(5) ☐
**Tai-chi, Qi gong?**	(1) ☐	(2) ☐	(3) ☐	(4) ☐	(5) ☐
**Yoga?**	(1) ☐	(2) ☐	(3) ☐	(4) ☐	(5) ☐
**Homeopathy?**	(1) ☐	(2) ☒	(3) ☐	(4) ☐	(5) ☐
**Aromatherapy?**	(1) ☐	(2) ☐	(3) ☐	(4) ☐	(5) ☐
**Relaxation therapy (Sophrology)?**	(1) ☐	(2) ☐	(3) ☐	(4) ☐	(5) ☐
**Healer/secret ?**	(1) ☐	(2) ☐	(3) ☐	(4) ☐	(5) ☐
**Other?** **Specify__________________**	(1) ☐	(2) ☐	(3) ☐	(4) ☐	(5) ☐
