# Supplementary material for: Complementary medicine among individuals experiencing homelessness in Switzerland: a quantitative and qualitative descriptive study
Source: BMC Complement Med Ther. 2025 May 6;25:166. doi: 10.1186/s12906-024-04727-4 (PMC12057206; doi:10.1186/s12906-024-04727-4)
Supplement: Supplementary file 2 — Supplementary Material 2 [file 12906_2024_4727_MOESM2_ESM.pdf]

## Appendix 1. Semi-structured interview guides

| Semi-structured interview guide with PEH | | |
|---|---|---|
| **Interests and expectations of complementary medicine approaches** | **[Use the answers to the quantitative questionnaire on complementary approaches as a basis for further study]**<br><br>**What do you think of complementary medicine approaches such as homeopathy, osteopathy, hypnosis and relaxation?**<br>• Interests<br>• Beliefs<br><br>**What complementary medicine approaches have you already tried in your life?**<br>• Why (to treat what)?<br>• What did you think? How have you benefited from it?<br><br>**What kind of complementary medicine did you grow up with, which is part of your culture?**<br>• Description<br>• Current access<br><br>**What approaches would you like to use now?**<br>• Why?<br>• What would that get you?<br>• Access, barriers? | ***Describe the interests and expectations of complementary approaches*** |
| **Opinions and recommendations on the intervention to be developed** | **As a follow-up to this project, we plan to develop a program offering a complementary approach for interested people who experience homelessness.**<br><br>**What do you think this intervention should be used for?**<br>• Objectives (improve what in terms of health or else)<br><br>**What approach(es) do you think we should develop?**<br><br>**Why?**<br><br>**Who should give this intervention?**<br><br>**Where?**<br>**What would motivate to take part of it?**<br><br>**In the contrary, what would or discourage you from taking part?**<br><br>**What else would you like to share?** | ***Describe advice and recommendations on how to develop the intervention***<br><br>***Conclude the interview and thank the participant for his/her time*** |

| Semi-structured interview guide with professionals | | |
|---|---|---|
| **Perceptions of the usefulness and expectations of complementary medicine approaches in people experiencing homelessness** | **In your opinion, to what extent do complementary medicine approaches such as homeopathy, osteopathy, hypnosis or relaxation techniques meet the needs of people experiencing homelessness?**<br>• Usefulness<br>• Interests<br>• Beliefs<br>• Using these approaches<br><br>**In your experience, what complementary medicine approaches are used by this population?**<br>• What are the reasons for this?<br>• Where ?<br><br>What obstacles do you see to the use of these approaches among people experiencing homelessness?<br><br>**What approaches do you think could help them?**<br>• Why? | ***Describe the perceived interests and expectations of complementary approaches in PEH*** |
| **Opinions and recommendations on the intervention to be developped** | **As a follow-up to this project, we plan to develop a program offering a complementary medicine approach for interested people experiencing homelessness.**<br><br>**What do you think this intervention should be used for?**<br>• Objectives (improve what in terms of health)<br><br>**What approach(es) do you think we should develop?**<br><br>**Why?**<br><br>**Who should give this intervention?**<br><br>**Where?**<br><br>**In your opinion, what should we do to encourage PEH to take part in the intervention?**<br><br>**Similarly, what should we do to limit the risk of disengagement during the intervention (how do we retain users over time)?** | ***Describe advice and recommendations on how to develop the intervention*** |
| | **What other elements would you like to share?** | ***Conclude the interview and thank the participant for his/her time*** |
